# The Expression of Snail, Galectin-3, and IGF1R in the Differential Diagnosis of Benign and Malignant Pheochromocytoma and Paraganglioma

**DOI:** 10.1155/2020/4150735

**Published:** 2020-02-27

**Authors:** Liling Deng, Tao Chen, Huan Xu, Yuanmei Li, Mingyan Deng, Dan Mo, Haoming Tian, Yan Ren

**Affiliations:** ^1^Department of Endocrinology and Metabolism, West China Hospital of Sichuan University, Chengdu, Sichuan 610041, China; ^2^Department of Endocrinology and Nephrology, Chongqing Emergency Medical Center (Chongqing the Fourth Hospital), No. 1 Jiankang Road, Yuzhong District, Chongqing 400014, China; ^3^Department of Pathology, West China Hospital of Sichuan University, Chengdu, Sichuan 610041, China

## Abstract

**Objective:**

The aim of this study was to investigate the expression of Snail, galectin-3, and IGF1R in benign and malignant pheochromocytoma and paraganglioma (PPGL) and explore their role in the diagnosis of malignant PPGL.

**Methods:**

We retrospectively collected and analyzed surgical tumor tissue from 226 patients initially diagnosed with PPGL who underwent surgery from Jan. 2009 to Jan. 2016 at West China Hospital, Sichuan University. We observed and quantified the expression of Snail, galectin-3, and IGF1R in paraffin-embedded samples by immunohistochemical staining.

**Results:**

The significant difference in survival time among the three groups (benign PHEO, benign PGL, and potentially malignant PPGL) was compared by Kaplan-Meier survival analysis. The positive staining of Snail, galectin-3, and IGF1R in the benign PHEO group was significantly lower than that in the other three groups (*P* < 0.001). The Kaplan-Meier survival plots indicated that the survival time of the patients with intense positive staining was significantly lower than that of the patients with weak positive staining.

**Conclusion:**

The intense expression of Snail, galectin-3, and IGF1R may be valuable indicators for the diagnosis of malignant PPGL.

## 1. Introduction

Pheochromocytoma and paraganglioma (PPGL) are rare tumors arising from chromaffin cells that commonly produce one or more catecholamines, including epinephrine, norepinephrine, and dopamine. These tumors are rarely biochemically silent. Approximately 85% of chromaffin-cell tumors are pheochromocytomas (PHEOs), whereas 15% are paragangliomas (PGLs) [1]. Approximately 10% of PHEOs and 30% of PGLs will undergo malignant transformation [2]. According to the guidelines, malignancy is defined as the presence of metastases in nonchromaffin tissue, and the prevalence varies between 10 and 17% [3]. The prognosis of malignant PPGL is very poor. However, currently, only few histological or biochemical markers are available to differentiate malignant PPGLs in the limited literature. Studies are needed to identify new biomarkers that can distinguish malignant PPGL during the early stage.

Snail, a vital regulator of neural crest migration, regulates the migration of chromaffin cells during the formation of normal adrenal glands [4]. Snail is also involved in the epithelial-to-mesenchymal transition (EMT). EMT explains how tumor cells escape from the primary tumor via Snail, which plays a vital role in tumor metastasis. In the current models of tumorigenesis, stemness and the tumor microenvironment are considered retroactive factors responsible for metastasis formation, and Snail serves as a regulator of these metastatic forces [5]. Hayry et al. found a high frequency of Snail-expressing tumor cells in primary PHEO, which suggested metastatic potential in 42 patients with PHEO [6].

Galectin-3 is a member of the *β*-galactoside-binding lectin family that is involved in multiple stages of cancer progression and metastasis [7]. Galectin-3 has been recognized as a sensitive and reliable diagnostic approach for the preoperative identification of thyroid carcinomas [8]. The intense expression of galectin-3 has also been observed in lung, breast, colorectal, and prostate carcinomas [9, 10]. Moreover, Gimm et al. first showed distinct galectin-3 staining patterns in various types of PHEO with small sample sizes [11].

IGF1R plays an important role in the development and function of many normal tissues. Accumulating evidence has revealed the important role of IGF1R in proliferative and antiapoptotic events. IGF1R in tumor tissue and its relevance in tumor growth and metastasis have been demonstrated in several types of cancer, such as breast cancer and pancreatic cancer [12, 13]. Fernandez et al. studied the labeling of IGF1R in 40 patients with primary PPGL and found a strong association between the increased expression of IGF1R and malignant PHEO [14].

Considering the probable role of these three biomarkers in the differentiation of malignant PPGL, we retrospectively collected and analyzed tumor tissues from 226 patients initially diagnosed with PPGL, investigated the expression of Snail, galectin-3 and IGF1R in benign and malignant PPGL, and further explored their role in the diagnosis of malignant PPGL.

## 2. Materials and Methods

### 2.1. Patient Selection

A total of 226 patients initially diagnosed with PPGL who underwent surgery between Jan. 2009 and Jan. 2016 at the West China Hospital of Sichuan University were included in the study. All patients had complete clinical and pathological data and were followed up until Jan. 2017. Malignancy is defined as the presence of metastases in nonchromaffin tissue. Based on the biological behavior, clinical outcome, and locations of these tumors, the 226 cases were divided into the following 4 groups. Tumors located in adrenal glands without local invasion or metastasis of nonchromophobic tissue were defined as benign PHEOs. Those ex-adrenal glands without local invasion or metastasis were defined as benign PGLs. PHEOs or PGLs with metastasis were defined as malignant PPGLs. Several PPGLs with invasion of the local blood vessels, lymph node, and peripheral adipose tissue had a higher risk of nonchromophobic tissue metastasis and were, thus, recognized as underlying malignancy in our study. In total, 96 benign PHEOs, 38 benign PGLs, 14 metastatic PPGLs, and 78 PPGLs of potential malignancy were included in the study. Paraffin-embedded tumor tissues were collected from eligible patients for immunohistochemistry analysis.

The protocol was approved by the Ethical Committee of the West China Hospital of Sichuan University. All participants provided written informed consent.

### 2.2. Statistical Analysis

SPSS 21.0 software was used for all statistical analyses. For the categorical variables, the data were analyzed using the *X*^2^ test; if the *X*^2^ test could not be used, Fisher's exact test was chosen. An analysis of receiver operating characteristic (ROC) curves was performed to evaluate the diagnostic performance of Snail, galectin-3, and IGF1R in malignant PPGL. Kaplan-Meier survival plots were constructed to determine whether the expression of Snail, galectin-3, and IGF1R influenced tumor metastasis. A *P* value less than 0.05 was considered statistically significant.

### 2.3. Immunohistochemistry

Paraffin-embedded tumor tissues were selected for the immunohistochemistry (IHC) studies. Sections (3 *μ*m thick) from representative blocks of each tumor were deparaffinized with xylene and alcohol and then rehydrated. The slides were immersed in 3% hydrogen peroxide for 15 minutes. The antigen retrieval was performed at an appropriate pH for each marker. Subsequently, the sections were incubated with a primary antibody for 1 hour and then washed and incubated with a secondary antibody for 30 minutes. A commercially available antibody against Snail (Abcam, UK) was used at a dilution of 1 : 100. An antibody against galectin-3 (CST, USA) was used at a dilution of 1 : 400. An antibody against IGF1R (CST, USA) was used at a dilution of 1 : 500. Finally, the antigens were detected by using a chromogen. Benign adrenal cortical adenomas were used as negative controls, breast carcinoma was used as a positive control for IGF1R, and lung carcinoma was used as a positive control for Snail and galectin-3. This study used the Barnes semiquantitative integration method to judge the staining results as follows: 5 high-power fields were randomly selected for counting; 0 points were assigned if the percentage of positive cells was 0-5%, 1 point was assigned if the percentage of positive cells was 6-25%, 2 points were assigned if the percentage of positive cells was 26-50%, 3 points were assigned if the percentage of positive cells was 51-75%, and 4 points were assigned if the percentage of positive cells was 76-100%. The immunostaining intensity was scored as follows: 0 points for no staining, 1 point for light yellow particles, 2 points for brown particles, and 3 points for tan particles. The two scores were multiplied to calculate the final results. The staining was scored as negative (0 points), weakly positive (1-4 points), intermediately positive (5-8 points), or strongly positive (9-12 points). The staining was scored by two independent readers.

## 3. Results

### 3.1. Basic Characteristics

The patients in this study were divided into 4 groups. The clinical characteristics of each group are summarized in Table 1. The patients with benign PPGLs were older than those with malignant PPGLs. No differences were observed in sex, plasma norepinephrine levels, epinephrine levels, tumor diameter, or tumor behavior among the groups.

### 3.2. Follow-Up Data

**Figure 1:**
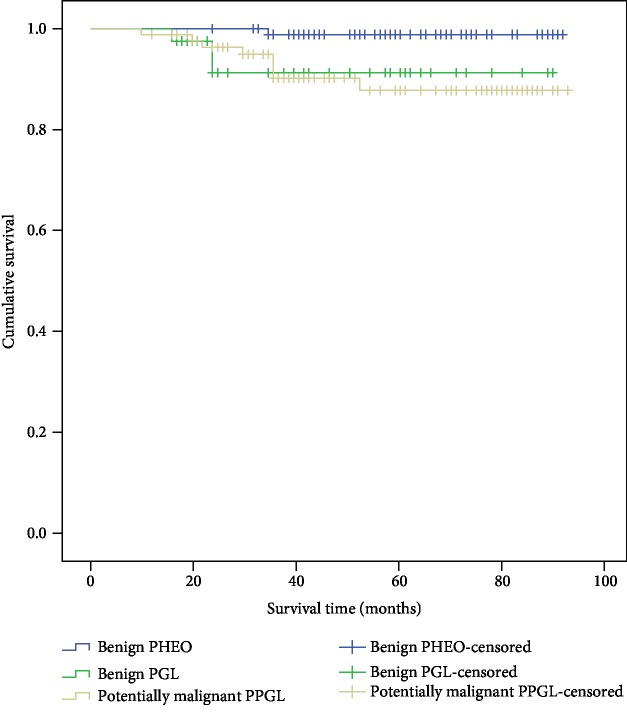
Cumulative Kaplan-Meier survival curves.

Among all 226 patients, 2 patients were diagnosed with malignant PPGL at the initial pathologic evaluation and the remaining 224 cases were divided into 3 groups (benign PHEO, benign PGL, and potentially malignant PPGL) and followed until Jan. 2017 to obtain the clinical outcomes. PPGL with pathological invasion of the local blood vessels, lymph node and fatty infiltration, or tumor necrosis was regarded as potentially malignant. The significant difference in survival time among the three groups was observed by a Kaplan-Meier survival analysis (Figure 1).

### 3.3. Expression of Snail, Galectin-3, and IGF1R in PPGLs

**Figure 2:**
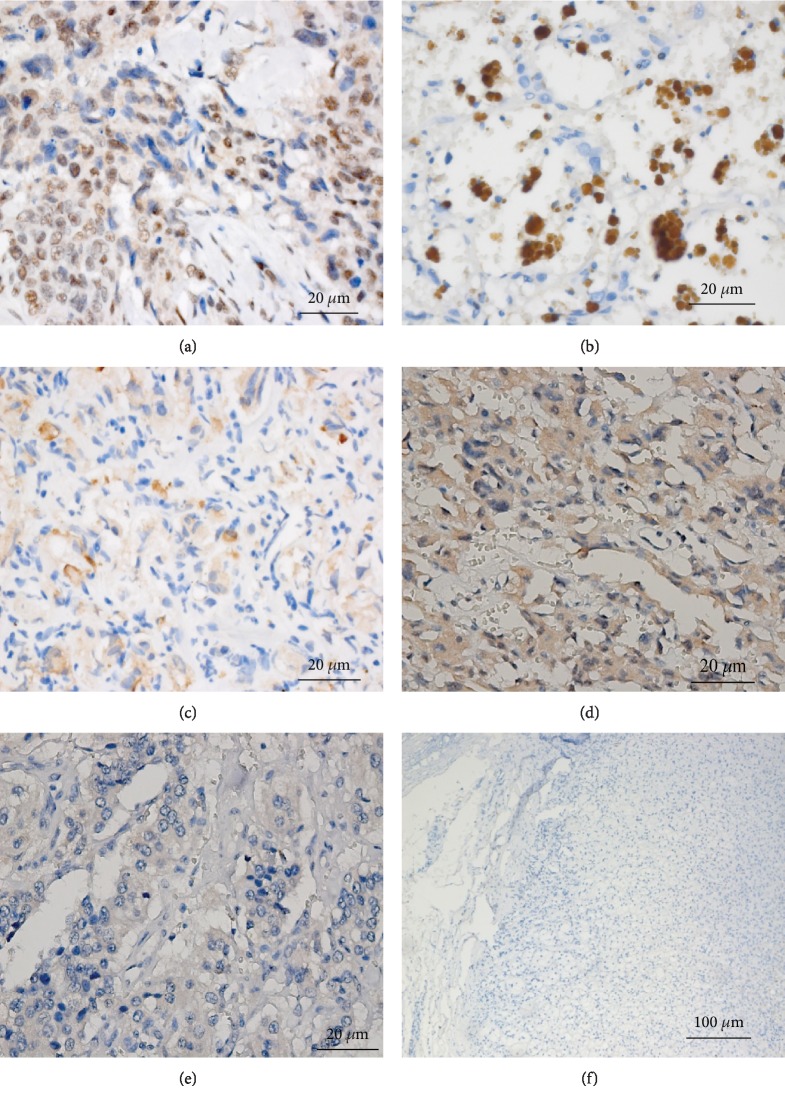
Snail immunostaining in PHEO and PGL. (a) Intense staining of Snail in positive control tissue (breast carcinoma) (Zeiss imager ×400). (b) Intense nuclear staining of Snail in PPGL tumor tissue (Zeiss imager ×400). (c) Weak nuclear staining of Snail in PPGL tumor tissue (Zeiss imager ×400). (d) Intermediate nuclear staining of Snail in PPGL tumor tissue (Zeiss imager ×400). (e) Negative nuclear staining of Snail in PPGL tumor tissue (Zeiss imager ×400). (f) Negative nuclear staining of Snail in normal adrenal glands (Zeiss imager ×100).

**Figure 3:**
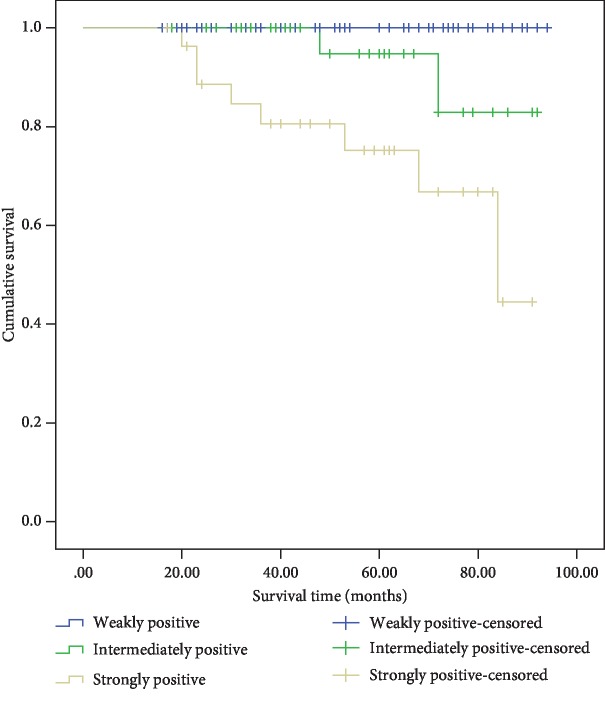
Cumulative Kaplan-Meier survival curves—Snail immunostaining intensity.

Snail expression in the 226 specimens was assessed by IHC (Figure 2). Among the 96 benign PHEOs, 47.5% exhibited positive staining. Of the 38 PGLs, 65.8% were positive. Of the 78 potentially malignant PPGLs, 80.8% were positive. Of the 14 malignant PPGLs, 78.6% were positive. The positive staining of Snail in the benign adrenal PHEO group was significantly lower than that in the other three groups (*P* < 0.001). Intense expression of Snail was predominant in the cases of confirmed malignant PPGLs. Based on positive Snail staining alone, the sensitivity and specificity for diagnosing malignant PPGL were 84.2% and 79.5%, respectively (Table 2). The Kaplan-Meier survival plots indicated that the survival time of the patients with intense positive staining was significantly lower than that of the patients with weak positive staining (Figure 3).

**Figure 4:**
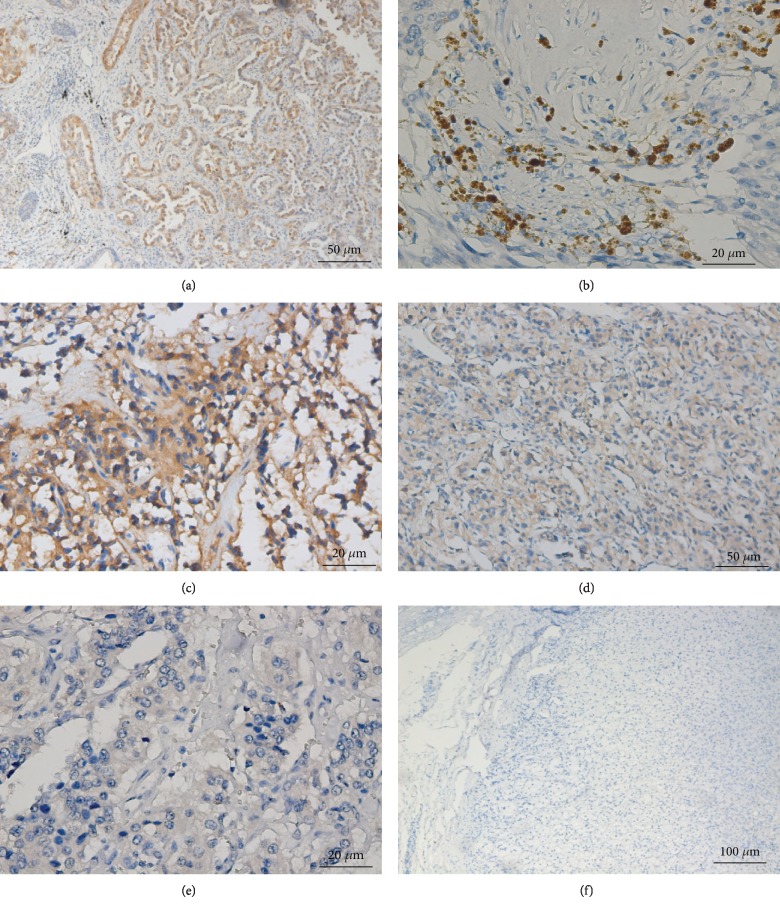
Galectin-3 immunostaining in PHEO and PGL. (a) Intense staining of galectin-3 in positive control tissue (lung carcinoma) (Zeiss imager ×200). (b) Intense cytoplasm staining of galectin-3 in PPGL tumor tissue (Zeiss imager ×400). (c) Intermediate cytoplasm staining of galectin-3 in PPGL tumor tissue (Zeiss imager ×400). (d) Weak cytoplasm staining of galectin-3 in PPGL tumor tissue (Zeiss imager ×200). (e) Negative cytoplasm staining of galectin-3 in PPGL tumor tissue (Zeiss imager ×100). (f) Negative cytoplasm staining of galectin-3 in normal adrenal glands.

**Figure 5:**
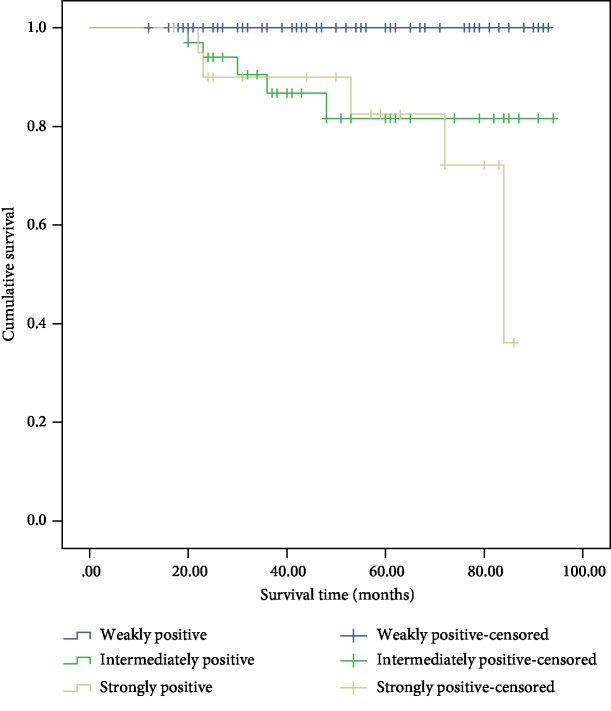
Cumulative Kaplan-Meier survival curves—galectin-3 immunostaining intensity.

Galectin-3 expression in the specimens was assessed by IHC (Figure 4). The positive expression of galectin-3 in benign PHEO was significantly lower than that in the other three groups (*P* < 0.001). Based on the positive galectin-3 expression alone, the sensitivity and specificity for diagnosing malignant PPGL were 77.3% and 79.5%, respectively (Table 3). The Kaplan-Meier survival analysis showed that the patients with intense positive staining had a shorter survival time than the patients with weak positive staining (Figure 5).

**Figure 6:**
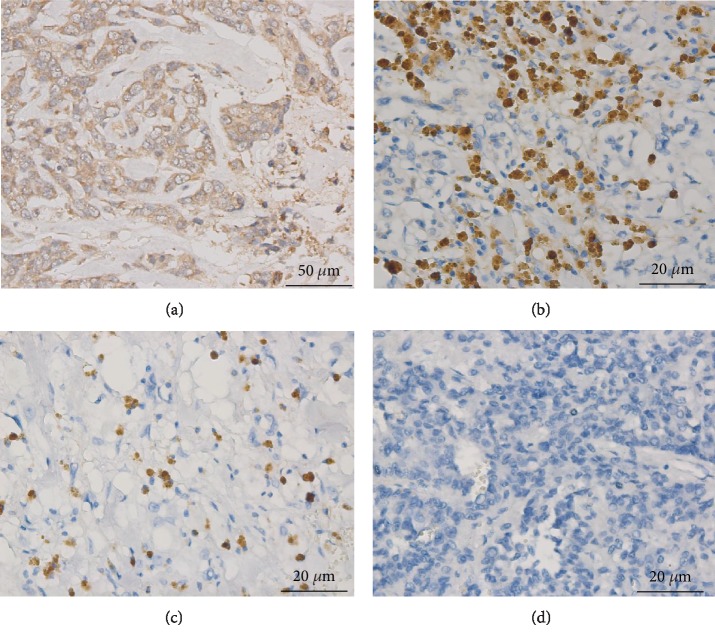
IGF1R immunostaining in PHEO and PGL. (a) Intense cell membrane and cytoplasm staining of IGF1R in positive control tissue (breast carcinoma) (Zeiss imager ×200). (b) Intense cytoplasm staining of IGF1R in PPGL tumor tissue (Zeiss imager ×400). (c) Intermediate cytoplasm staining of IGF1R in PPGL tumor tissue (Zeiss imager ×400). (d) Negative cytoplasm staining of IGF1R in PPGL (Zeiss imager ×400).

**Figure 7:**
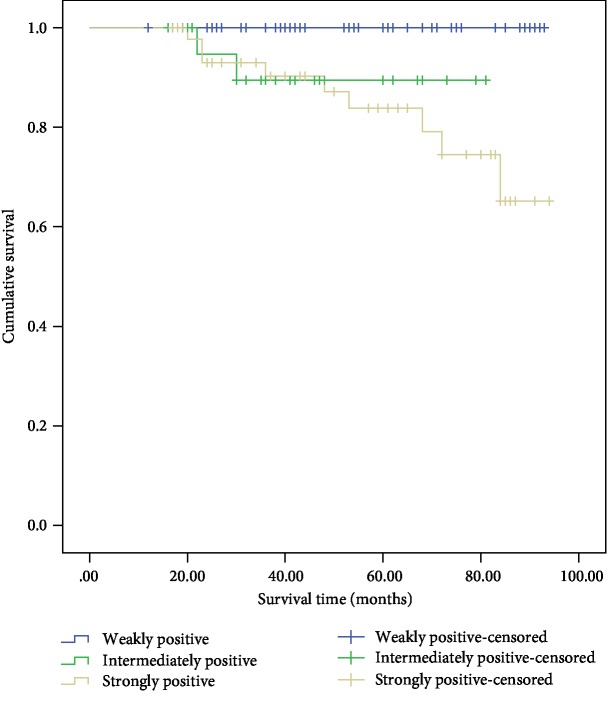
Cumulative Kaplan-Meier survival curves—IGF1R immunostaining intensity.

IGF1R expression in the specimens was assessed by IHC (Figure 6). The positive staining of IGF1R in benign PHEO was significantly lower than that in the other three groups (*P* < 0.001). Based on the positive IGF1R staining alone, the sensitivity and specificity for diagnosing malignant PPGL were 89.5% and 75%, respectively (Table 4). The Kaplan-Meier survival analysis indicated that the survival time of the patients with intense positive staining was significantly lower than that of the patients with weak positive staining (Figure 7).

## 4. Discussion

Approximately 10% of PHEOs and 30% of PGLs undergo malignant transformation [2]. Unfortunately, no reliable histological or biochemical markers are available to differentiate benign from malignant PPGL. Currently, malignant behavior is defined only by the appearance of metastasis. The early diagnosis of malignant PPGL is difficult in clinical practice. The pathological characteristics of malignant tumors, such as multiple pathological mitotic figures, tumor necrosis, and the capsule or blood vessels or surrounding fatty infiltration, were not confirmed as the gold standard of malignant PPGLs. Malignancy is defined as the presence of metastases in nonchromaffin tissue [3]. In addition, blood vessel invasion or tumor thrombus is a risk factor for malignancy in PPGL [15].

In recent years, the diagnostic value of some pathological biomarkers has been evaluated in malignant PPGLs; however, the clinical significance remains unknown. The association between Snail and several carcinomas, such as renal cell carcinomas, has been demonstrated in numerous studies [5, 16, 17]. Snail clearly contributes to tumor progression in a much more potent manner by regulating the plasticity of tumor and tumor-activated cells and their crosstalk [18]. The role of Snail in the poor prognosis of malignant PPGLs has been addressed in a few studies with small sample sizes [19]. Here, the positive staining of Snail in the benign PHEO group was significantly lower than that in the other three groups (benign PGL, potentially malignant PPGL, and malignant PPGL) by IHC analysis. Based on the positive Snail staining alone, the sensitivity and specificity for diagnosing malignant PPGL were 84.2% and 79.5%, respectively. We divided the participants into groups based on the intensity of the positive labeling, and the Kaplan-Meier survival plots indicated that the risk of metastasis was much higher if Snail labeling in the tumor tissue was intense.

Intracellular galectin-3 is an antiapoptotic factor involved in homotypic aggregation. Furthermore, the tumor-endothelial cell interactions required for metastasis are believed to be mediated by endothelium-associated galectin-3 [7]. Galectin-3 plays an important role in tumor progression and metastasis in different types of tumors, such as lung cancer and prostate cancer [7, 20]. Additionally, galectin-3 is highly specific for thyroid malignancy [21, 22]. In recent years, higher expression of galectin-3 in malignant PPGL has been indicated as a possible histological marker to differentiate malignant from benign PPGL in a few studies involving a small group of participants [23]. Thus, this association must be assessed in a large cohort of patients in the near future. In this study, the positive expression of galectin-3 in the benign adrenal PHEO group was significantly lower than that in the other three groups by IHC analysis. The sensitivity and specificity for malignant PPGL diagnosis were 77.3% and 79.5%, respectively. Moreover, a worse outcome in PPGL patients was associated with intense galectin-3 expression levels as shown by the Kaplan-Meier analysis. Therefore, the intense expression of galectin-3 may be a valuable indicator for the diagnosis of malignant PPGL. Therefore, PPGL patients with a high expression of galectin-3 should be closely followed up after surgery.

IGF-1R triggers a signaling cascade leading to proliferative and antiapoptotic events [24]. The relevance of IGF1R in tumor tissue, tumor growth, and metastasis has been demonstrated in several types of cancer, such as breast cancer and pancreatic cancer [12, 13]. Fernandez et al. studied the labeling of IGF1R in 40 subjects with primary PPGL and found a strong association between the increased expression of IGF1R and malignant familial PHEO/PGL regardless of the genetic etiology [14]. In our study, the expression of IGF1R in benign PHEO was significantly lower than that in malignant PPGL by the IHC analysis. Based on the positive IGF1R labeling alone, the sensitivity and specificity for diagnosing malignant PPGL were 89.5% and 75.0%, respectively. The risk of metastasis was much higher with strongly intense IGF1R expression by the Kaplan-Meier survival analysis. Thus, we speculate that IGF1R expression could be a reliable marker for differentiating and predicting metastasis in PPGLs.

The present study has several limitations. First, in our study, the average follow-up time was 4.6 years, while the longest follow-up time was 7.8 years. Some cases with high intensity staining were followed for less than the average number of years, and these cases may become malignant in the following years. In addition, other patients classified as bearing PPGLs of potential malignancy may indeed never develop malignancy despite old age. Thus, a longer follow-up should be needed to detect more malignant cases, indicating that patients with high intensity IGF1R expression should be followed up more frequently. Numerous studies are required to establish the role of these three markers in the differential diagnosis of benign and malignant PPGLs.

Second, several paraffin-embedded tumor tissues were preserved for a long time at room temperature from eligible patients and were collected for the IHC analysis. The protein in these tumors may have broken down over time, leading to lower positive staining in this study.

Furthermore, in the reported literature, up to 50% of patients with metastatic PPGLs carry hereditary germline mutations, mainly in the SDHB gene [25]. SDHB-mutated PPGs activate the process of epithelial-to-mesenchymal transition (EMT), which plays a vital role in tumor metastasis. Therefore, a comparison of SDHB and various IHC makers in PPGLs needs to be performed in the future.

In conclusion, the intense expression of Snail, galectin-3, and IGF1R may be valuable indicators for the diagnosis of malignant PPGL. PPGL patients with a high expression of Snail, galectin-3, and IGF1R should be closely followed up after surgery.

## Figures and Tables

**Table 1:** Clinical characteristics of the study patients.

	Benign PHEO (*n* = 96)	Benign PGL (*n* = 38)	Potentially malignant PPGL (*n* = 78)	Malignant PPGL (*n* = 14)	*P*
Gender (male/female)	50/46	18/20	28/50	6/8	0.269
Age	49.63 ± 14.83	53.11 ± 13.69	45.43 ± 14.68	39.35 ± 15.34	0.008
Tumor size (cm)	5.0 (3.5-7.0)	5.0 (4.0-6.0)	5.0 (4.0-8.0)	6.5 (3.25-10.5)	0.158
Blood NE (ng/L)	1198.0 (543.5-3948.0)	1343.5 (662.5-2815.5)	2603 (1000-6004)	1129.5 (646.3-378)	0.081
Blood E (ng/L)	69.5 (41.6-226.3)	74 (48-200)	103.5 (50-376.3)	61 (50-94)	0.228

**Table 2:** Snail IHC expression in PPGLs.

	Negative (*n* (%))	Weakly positive (*n* (%))	Intermediately positive (*n* (%))	Strongly positive (*n* (%))	*X* ^2^	*P*
Benign PHEO (*n* = 96)	60 (62.5)	30 (31.3)	5 (5.2)	1 (1.0)		
Benign PGL (*n* = 38)	13 (34.2)	11 (28.9)	8 (21.1)	6 (15.8)	37.121	<0.001
Potentially malignant PPGL (*n* = 78)	15 (19.2)	32 (41.0)	18 (23.1)	13 (16.7)		
Malignant PPGL (*n* = 14)	3 (21.4)	0 (0)	2 (14.3)	9 (64.3)		

**Table 3:** Galectin-3 IHC expression in PPGLs.

	Negative (*n* (%))	Weakly positive (*n* (%))	Intermediately positive (*n* (%))	Strongly positive (*n* (%))	*X* ^2^	*P*
Benign PHEO (*n* = 96)	72 (75.0)	22 (22.9)	2 (2.1)	0 (0)		
Benign PGL (*n* = 38)	10 (26.3)	14 (36.8)	12 (31.6)	2 (5.3)	76.741	<0.001
Potentially malignant PPGL (*n* = 78)	13 (16.7)	34 (43.6)	17 (21.8)	14 (17.9)		
Malignant PPGL (*n* = 14)	1 (7.1)	0 (0)	6 (42.9)	7 (50)		

**Table 4:** IGFR1 IHC expression in PPGLs.

	Negative (*n* (%))	Weakly positive (*n* (%))	Intermediately positive (*n* (%))	Strongly positive (*n* (%))	*X* ^2^	*P*
Benign PHEO (*n* = 96)	73 (76.0)	22 (22.9)	1 (1.1)	0 (0)		
Benign PGL (*n* = 38)	13 (34.2)	8 (21.1)	6 (15.8)	11 (28.9)	75.693	<0.001
Potentially malignant PPGL (*n* = 78)	12 (15.4)	24 (30.8)	14 (17.9)	28 (35.9)		
Malignant PPGL (*n* = 14)	2 (14.3)	0 (0)	3 (21.4)	9 (64.3)		

## Data Availability

The data used to support the findings of this study are included within the article.
